# Mechanosensation and Mechanotransduction in Natural Killer Cells

**DOI:** 10.3389/fimmu.2021.688918

**Published:** 2021-07-15

**Authors:** Giorgio Santoni, Consuelo Amantini, Matteo Santoni, Federica Maggi, Maria Beatrice Morelli, Angela Santoni

**Affiliations:** ^1^ School of Pharmacy, Section of Experimental Medicine, University of Camerino, Camerino, Italy; ^2^ School of Biosciences and Veterinary Medicine, University of Camerino, Camerino, Italy; ^3^ Macerata Hospital, Oncology Unit, Macerata, Italy; ^4^ Department of Molecular Medicine, Sapienza University, Rome, Italy; ^5^ IRCCS Neuromed, Pozzilli, Italy

**Keywords:** mechanosensation, mechanotransduction, natural killer (NK) cells, immunological synapse, cytotoxicity

## Abstract

Natural killer (NK) cells are a main subset of innate lymphocytes that contribute to host immune protection against viruses and tumors by mediating target cell killing and secreting a wide array of cytokines. Their functions are finely regulated by a balance between activating and inhibitory receptors and involve also adhesive interactions. Mechanotransduction is the process in which physical forces sensed by mechanosensors are translated into chemical signaling. Herein, we report findings on the involvement of this mechanism that is mainly mediated by actin cytoskeleton, in the regulation of NK cell adhesion, migration, tissue infiltration and functions. Actin represents the structural basis for NK cell immunological synapse (NKIS) and polarization of secretory apparatus. NK-target cell interaction involves the formation of both uropods and membrane nanotubes that allow target cell interaction over long distances. Actin retrograde flow (ARF) regulates NK cell signaling and controls the equilibrium between activation *versus* inhibition. Activating NKIS is associated with rapid lamellipodial ARF, whereas lower centripetal actin flow is present during inhibitory NKIS where β actin can associate with the tyrosine phosphatase SHP-1. Overall, a better knowledge of mechanotransduction might represent a future challenge: Realization of nanomaterials tailored for NK cells, would be important to translate *in vitro* studies in *in vivo* new immunotherapeutic approaches.

## Natural Killer (NK) Cell Target Recognition and Functions

Natural Killer (NK) cells represent the prototype of innate lymphoid cells that act as first line of defense against microbial infections, and tumor cell transformation, growth and metastatic spreading (1–3). NK cells prompt the response mainly on their ability to release lytic mediators, such as perforin and granzymes, or to express ligands triggering death receptors on target cells; moreover, they can secrete a wide array of cytokines and chemokines to recruit and educate other immune cell types (4, 5). NK cell activation depends on a delicate balance between activating and inhibitory signals, being the latter mainly transduced by killer-cell immunoglobulin-like receptors (KIRs), cluster of differentiation 94 (CD94)/natural-killer group 2, member A (NKG2A) receptors for class I MHC. Recognition of abnormal self on tumor or viral infected cells triggers a number of non MHC I-restricted activating receptors such as NKG2D, the receptor for the human MHC I-related sequence A and B (MICA/B) and UL16 binding proteins (ULBPs), DNAX accessory molecule-1 (DNAM-1) that recognizes nectin-2 (Nec2, CD112) and nectin-15 (Necl5, PVR, CD155), and the natural citotoxicity receptors (NCR) receptors (1). In addition, NK cells express receptors belonging to the β1 and β2 integrin family with lymphocyte function-associated antigen 1 (LFA-1) playing a central role in priming NK cells for cytotoxicity. The dialog of the β1 and β2 integrins with a number of chemokine receptors is also crucial for the homing and migration of NK cells in lymphoid and non-lymphoid organs.

Moreover, it is becoming clear that although they act in cooperative manner, different activating receptors can independently trigger discrete steps of NK cytolytic process (i.e. target cell contact and adhesion, granule polarization, degranulation and target lysis) by initiating diverse signaling cascades (6). In this regard, signaling components controlling cytoskeleton rearrangement, are emerging as critical events for NK cell cytotoxicity and migration.

NK/target cell interaction is regulated by adhesion molecules such as β1 and β2 integrins that play either a receptor or co-receptor role (7). High-avidity/affinity of integrins occurs in response of different activating receptors and depends on the activation of different signaling pathways including tyrosine kinases (PTK) belonging to the proto-oncogene tyrosine-protein kinase (Src) family, phosphoinositide 3-kinase (PI3K), small G proteins, and cytoskeletal integrity (inside-outside signaling). β2 integrins have been also shown to be critical in the interaction between NK and target cells by controlling the formation of the immunological synapse, which is an assembly of membrane receptors and signal transduction molecules largely driven by actin cytoskeleton. Development of NK cell cytotoxic functions requires the activation of a complex cascade of signaling pathways, including the activation of spleen tyrosine kinase (Syk) family PTKs, phospholipase C gamma (PLC-γ) and D (PLD), PI3K, Vav/Rac pathway, ERK, p38 and MAPKs (6). Some of these events are shared by different activating receptors, but distinct signals are also transduced depending on the type of receptor or the sensitive target triggering the cytotoxicity. In this regard, it has been reported that the activation of focal adhesion PTK Pyk2, that has been shown to be activated by β1 and β2 integrins, is a discriminating event between natural and antibody-mediated cytotoxicity. Moreover, ligation of integrins on human NK cells transduces intracellular signals leading to tyrosine phosphorylation of paxillin, intracellular calcium elevation (8), and co-stimulation of NK cell cytotoxic functions.

## Mechanosensing in the NK Cell-Mediated Immune Responses

Mechanotransduction is the process in which physical forces sensed by mechanosensors are translated into chemical signaling pathways. This mechanism mediated by the cytoskeleton that serves as a global mechanosensor apparatus, permits cells to sense their extracellular environment and rapidly respond to different stimuli. NK cell mechanosensors include a large range of activating receptors as well as β1 and β2 integrins and CD62L selectin (9, 10).The actomyosin network plays an important role in mechanotransduction, in that actin polymerization generates a “pushing” force, whereas the myosin produces a “pulling” force, and together are translated into several signaling cascades (11, 12). Thus, the actin cytoskeleton provides the mechanical forces necessary for adhesion, migration and tissue infiltration of NK cells as well as for their cytotoxic function. The actin interactome represents the structural basis for the formation of a stable NKIS, integration of molecular complexes and signaling components, and the secretion of cytolytic granules and mediators (e.g., perforin) leading to target cell killing. The mature activating NKIS contains a central and peripheral supramolecular activation cluster (SMAC). The β2 integrins, namely α_L_β2 (LFA-1) and α_M_β2 (Mac-1) and F-actin accumulate in the peripheral SMAC (pSMAC), whereas perforin is present in the central SMAC. The accumulation of F-actin and β2 integrins is rapid, it depends on Wiskott–Aldrich syndrome protein (WASp)-driven actin polymerization, and is not affected by microtubule depolymerization. Conversely, the polarization of perforin is slower and requires intact actin, WASp protein, and microtubule function (13–15).

## Uropods and Nanotubes Mediate Mechanotaxis in NK Cells

Cell guiding is involved in a number of biological processes, but however, its mechanisms remain still partially elucidated. Immune cells are able to migrate directionally thanks to chemical guiding or chemotaxis in response to chemo-attractants, to haptotaxis in response to surface-bound chemicals, and to mechanical guiding or mechanotaxis in response to mechanical stimuli such as substrate stiffness, cell deformation or osmotic stress (16).

A large body of evidences mainly regard chemotaxis, whereas mechanotaxis has been considered only recently. Although a role in the activation of leukocytes has been reported (17), the basic remain largely elusive. Immune cell trafficking is not only supported by chemical signals, but also involves mechanical signals like hydrodynamic shear stress (18–21). The external forces are perceived by leukocytes mainly through the integrin receptors, which undergo conformational changes by inside-out (22) and outside-in (23) signaling, and initiate an intracellular signaling cascade in response to mechanical forces. Thus, integrins are key players both in the tissue recruitment of leukocytes from blood flow, and in leukocyte mechanotaxis under flow.

Two types of orientation mechanisms by flow have been suggested to mediate leukocyte integrin-mediated adhesion. Shear stress involves integrin-mediated outside-in signaling at anchoring sites (24–26). Such mechanisms are considered “active”, in that a specific intracellular signaling pathway is initiated in response to flow, and relies on mechanotransduction. Alternatively, a “passive” model that does not require signaling by the external cue, has been also proposed for upstream crawling lymphocytes (27). In this model, flow direction is detected by the passive orientation uropod, which is not adherent and freely rotates. Reorientation of the whole cell against flow, follows tail orientation *via* the cell realignment by front-rear polarization. At molecular level, the cross-talk between LFA-1 and integrin very late antigen-4 (VLA-4, α4β1) and the opposite polarization of LFA-1 and VLA-4 integrins sustain a differential adhesion of leukocytes either by their leading or trailing edge.

NK cells circulate in the blood against fluid flow, as shown by the orientation of the non-adherent cell rear, the uropod. Uropods sense and transmit flow directions into cell steering through the polarity maintenance (27). In addition, NK cell migration involves the convergence of signaling events triggered by engagement of both β2 and β1 integrins, and a number of chemokine receptors including C–X–C motif chemokine receptor (CXCR) 4, C–C chemokine receptor type (CCR) 2, CCR5 and CXCR3 (28, 29).

Cell polarization is also a crucial event for the formation of NK/target cell conjugates. NK cells contact target cells exploiting the region with high concentration of CCR2 and CCR5, whereas the ligands of β2 integrin intercellular adhesion molecule (ICAM)-1 and ICAM-3, are concentrated at the distal pole in the uropods. Blocking cell polarization and adhesion receptor redistribution, inhibits NK cell cytotoxic activity as result of impaired effector–target cell conjugate formation. Thus, cell polarization regulates different steps of NK cell functions: the leading edge where the CCR are concentrated, is involved in the adhesion to target cells, polarization of the secretory apparatus and release of lytic granules during the NK cell killing (30, 31); remarkably, different types of lytic granules undergo polarized secretion to the site of membrane contact between NK and target cells (32). The uropods by accumulating ICAM molecules, are involved in the recruitment of NK cells.

Cell polarization and cytotoxic activity, can be blocked by adenosine diphosphate (ADP) ribosylation of the guanosine-5’-triphosphate (GTP) binding protein Ras homolog family member A (RhoA), indicating the important role of this signaling pathway triggered by LFA-1 as well as by CCR in these events (33, 34).

Notably, formation of NK/target cell conjugates also stimulates chemokine release, which can further promote NK cell binding to target cells by inducing integrin inside-outside signaling and support NK cell migration.

Beside the involvement of uropods in the regulation of the intimate NK cell contact with target cells, NK cells can also generate membrane nanotubes that allow these cells to functionally interact with targets over long distances. Target cells that relocate along the nanotube path, are polarized with their uropods facing the direction of movement, are then lysed. Removing the nanotubes by a micromanipulator reduces target cell lysis (35). The frequency of nanotube formation depends on the number of interactions between activating receptor and the respective ligand(s), and increases upon the NK cell activation. Imaging studies have demonstrated that proteins, such as the signaling adaptor that associates with NKG2D, DAP10, the G protein exchange factor Vav-1, and the NKG2D ligand MICA accumulate at nanotube synapses in a such high concentration to stimulate cell activation (35).

## NK Immunological Synapse (NKIS): Role of the Actin Cytoskeleton and Retrograde Flow in Controlling NK Cell Responses

As above mentioned, NK cell cytotoxicity is a tightly regulated multistep process. It moves through the initial contact between NK cells with target cells which is mediated by tethering receptors such as CD2 and the selectin CD62L, adhesive integrin receptors (LFA1 and Mac1) that interact with ICAM1, and activating receptors such as NCRs, NKG2D and DNAM1 (36). Both activating and integrin signaling initiate the formation of NKIS. In particular, engagement of β2 integrins, but not of CD-16 activating receptor on CD16.NK-92 cells, was found to affect the size and the dynamics of signaling microclusters in a Pyk2-dependent manner (37).

Signaling is required for cytoskeleton remodeling, formation of activating microclusters and adhesion ring junction, polarization of effector cells and cytolytic granule releases (34). Accumulation of F-actin at the NKIS is the pre-requisite for the clustering of activating receptors which stimulate the polarization of cytolytic granules to the immunological synapse (IS). Lytic granules navigate to actin meshwork at the IS and reach the plasma membrane through gaps sized to accommodate lytic granule movements. The movement of lytic granules is dependent to myosin IIa that generates force and movements along actin filaments. In NK cells, myosin IIa mediates the interaction between lytic granules and F-actin UNC45-dependent manner at the NKIS, and facilitates the movement of lytic cargo along actin filaments (38). The movement of lytic granules toward the IS, initially depends on dynein that mediates the movement of secretory vesicles to the MTOC (39), and then on the granule-associated small GTPase Rab27a that recruits the Slp3–kinesin-1 complex, thus enabling the polarized cytotoxic granules to reach the membrane and release their contents at the IS (9, 40, 41). Activating receptor-triggered secretion of lytic granules requires binding of Rab27a to Munc13-4, with formation of a complex that co-localizes with lytic granules (42). Lytic granules docked and tethered to NKIS, are primed through interaction between Munc 13-4 and STX11 to form a trans-soluble NSF attachment protein receptor (SNARE) complex comprising the syntaxin STX11 and synaptosomal SNAP23 protein together with the SNARE proteins vesicle associated membrane protein (VAMP) 4 and VAMP7, likely mediated by syntaxin-binding protein 2 (STXBP2) (43). After the fusion of lytic granules at the synaptic cleft, the lytic granule membrane proteins are endocytosed to replenish lytic granules for further killing.

**Figure 1:**
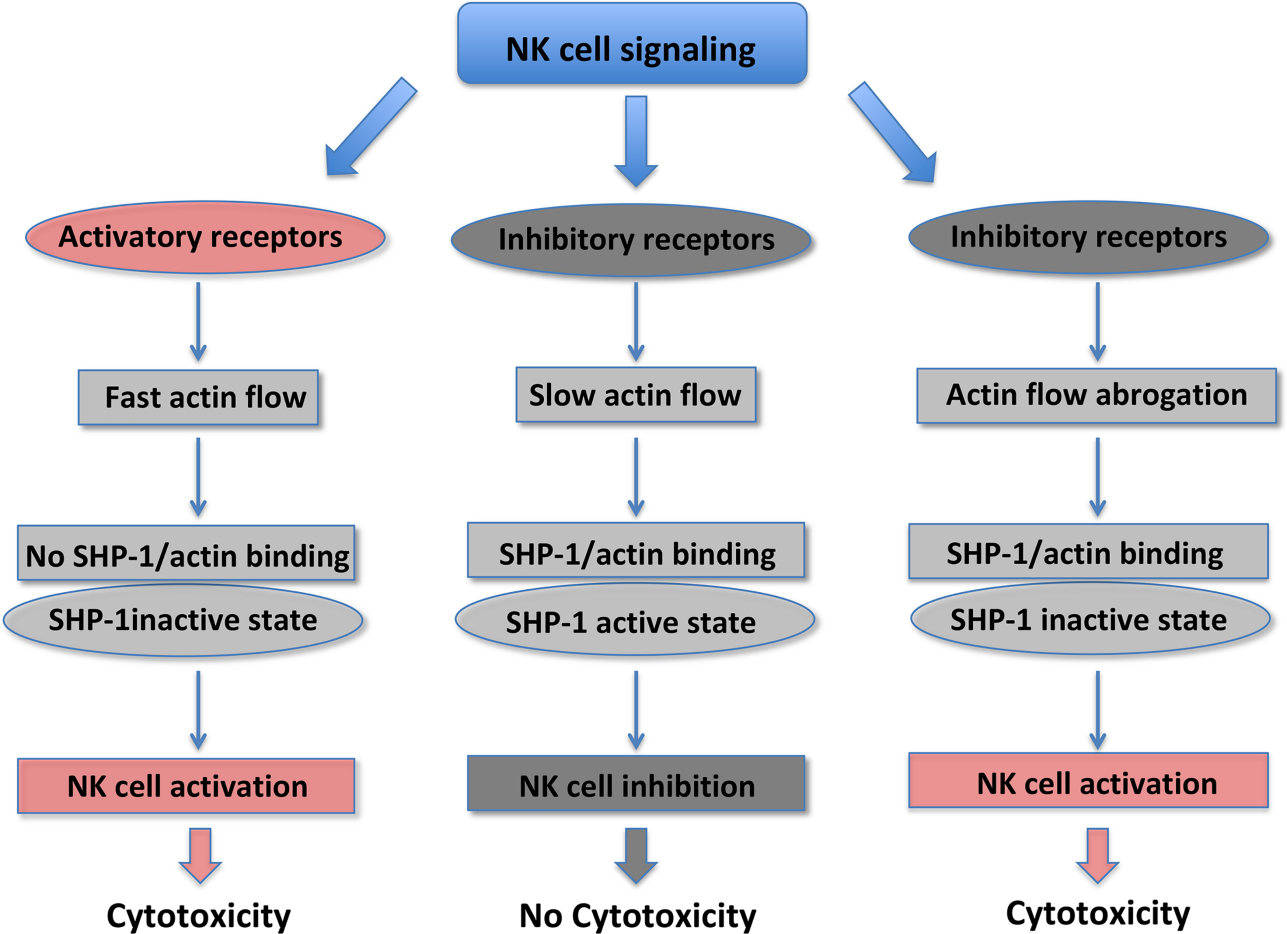
NK cell cytotoxicity is regulated by changes in SHP-1 conformational state.

F-actin is a major driver of the IS formation between NK and target cells, and the polarization of the secretory apparatus (44–46). Actin retrograde flow (ARF) regulates NK cell signaling and controls the equilibrium between NK cell activation *versus* inhibition. The actomyosin dynamics is responsible for the “mechanotransduction” (11, 12, 47). The F-actin turnover and myosin IIa force at the NKIS are the most important forces involved in the guide of the F-actin centripetal flow, with actin polymerization pushing at the external part of the spreading cell, and the contractile myosin force pulling the F-actin away from the cell membrane (48). It has been well accepted that the actin flow plays a pivotal role in the centralization of receptors and signaling proteins. Activating NKIS is associated with a rapid lamellipodial ARF, whereas a lower centripetal actin flow is present during inhibitory NKIS (49). It has been recently demonstrated that tyrosine protein phosphatase (SHP)-1 and β-actin specifically associate at the inhibitory NKIS where ARF is slower. In addition, SHP-1 is maintained in a closed status and catalytically inactive form, if actin flow is inhibited by using jasplakinolide or cytochalasin D pharmacological inhibitors, or by growing cells on rigid surfaces. Thus, SHP-1 inhibition is the result of actin flow reduction that leads to increased Vav-1 and PLCγ-1/2 tyrosine phosphorylation and to NK cell activation. SHP-1 does not interact with the actin machinery when ARF velocity is rapid and activating NK cell interactions occur; in this case it has a closed catalytically inactive conformation, allowing Vav-1 and PLC-γ1/2 tyrosine phosphorylation (**Figure 1**) (47, 50). Thus, stimulation of NK cell activating receptors induces a fast actin flow that inhibits the binding of SHP-1 with the actin network. This NK activating status favors SHP-1 closed inactive conformation promoting target cell killing. Engagement of KIR inhibitory receptors results in slow actin flow, enabling the formation of the SHP-1/β-actin complex. SHP-1 inhibits NK cell activation by dephosphorylating important signaling molecules. Given that the actin flow dynamics rapidly change, it may enable a rapid switching from an inhibitory to activating NK cell response (47).

NK cell inhibition is also mediated by SHP-2 recruitment to KIR receptors (51, 52), and in non-immune cells SHP-2 is associated with the actin cytoskeleton (53). Given that SHP-1 and SHP-2 display a high level of structural homology, it can be suggested that a similar regulation of SHP-2 occurs also in NK cells *via* dynamic actin movement.

Overall, these data indicate that actin mechanotransduction is essential to increase the NK cell capability to rapidly respond to local environmental changes. While NKIS formation requires minutes/hours and is a slow process, alterations of the actin flow dynamics and the following SHP-1 status are fast events (seconds/minutes) that permit a rapid on/off control of inhibitory signaling (47). Thus, ARF, by controlling actin dynamics, represents a novel mechanism to regulate the functional outcome of NK cells.

## Mechanical Properties of Surrounding Matrix and Targets Drive NK Cell Responsiveness

**Figure 2:**
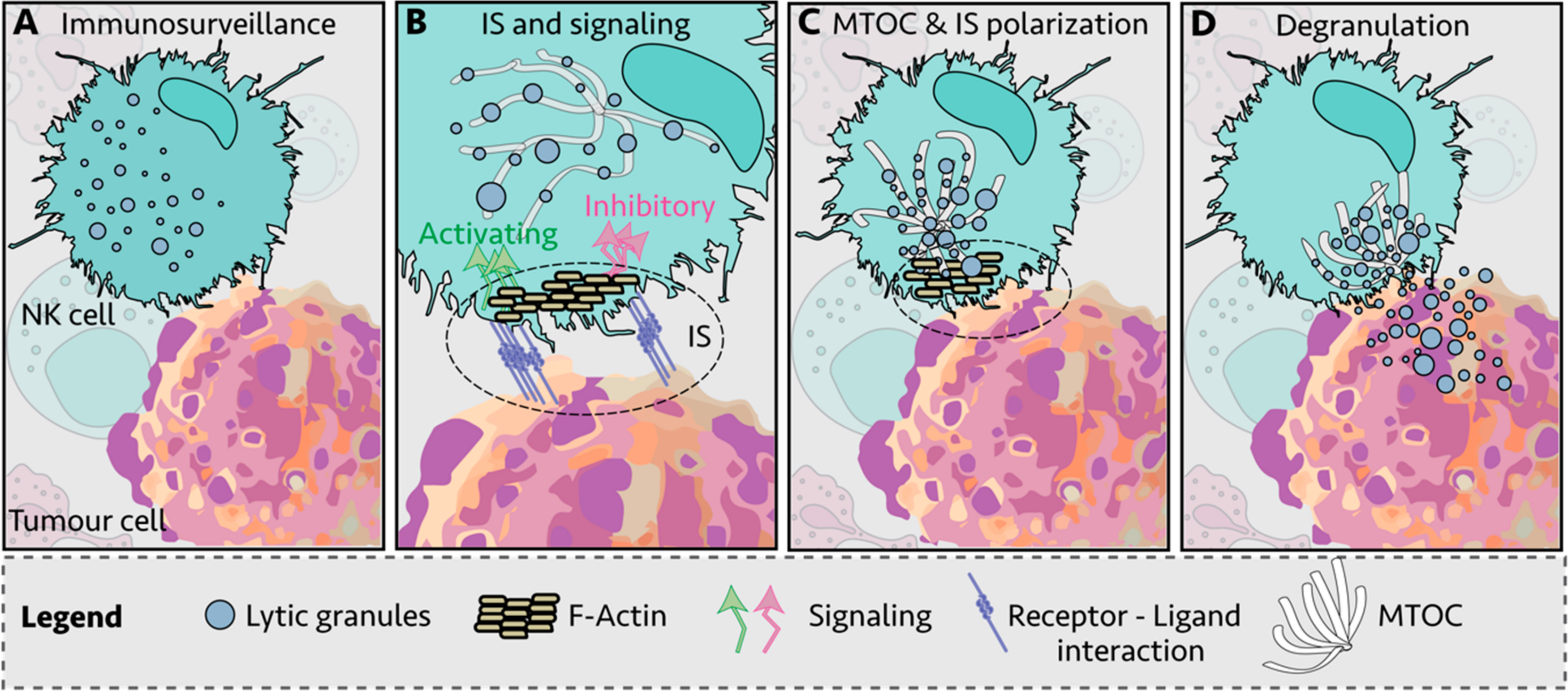
Immunological synapse (IS) of Natural Killer (NK) cell and target cell. **(A)** NK cells engage other cells to create an immunological synapse (IS); **(B)** First, filamentous actin (F-actin) is recruited to the IS; **(C)** NK lytic granules move along microtubules by dynein–dynactin motor proteins toward the microtubule-organizing center (MTOC); **(D)** the polarized lytic granules and MTOC dock at the IS, and degranulate (57).

During their lifetime, NK cells infiltrate a variety of normal, inflamed, or neoplastic tissues with different mechanical properties, exposing the NK cells to big fluctuations in matrix stiffness with potential impact on their responsiveness (54). Moreover, during the development, bone-marrow NK cells are exposed to substrate stiffness ranging from 25 to 100 kPa (55). Both NK cell degranulation and cytokine production are modified by the rigidity of the target substrate. In the study by Malaton and colleagues, the effect of substrate stiffness on efficiency of NK cell degranulation was evaluated by using a cell-sized beads coated with sodium alginate at defined soft (9 kPa), medium (34 kPa) or stiff (254 kPa) alone, or in the presence of antibodies directed against LFA-1 or the NCR receptor NKp30. Increased substrate stiffness stimulated NK cell degranulation, that was further enhanced by NKp30 and LFA1 triggering. The lower degranulating capacity of NK cells contacting soft targets was suggested to depend on an impaired recruitment of phosphorylated zinc-finger antiviral protein (Zap) 70 PTK to the NKIS (47). Similarly, a recent report demonstrated that target rigidity impacts on perforin granule polarization and degranulation, being secretion of granzymes A and B, granulysin and FAS ligand improved with increased substrate stiffness (56). Soft target stiffness was found to reduce F-actin accumulation and talin polarization and recruitment at the NKIS, leading to the formation of an instable asymmetrical synapse and decreased proportion of NK cells in the conjugates with targets. In addition, interaction with softer targets resulted in impaired microtubule organizing center (MTOC) and lytic granule polarization (**Figure 2**) (56).

Stiffness of the activating substrates not only impacts on NK cell cytotoxic function, but secretion of cytokines, namely interferon-gamma (IFN-γ), is also modulated, with increased release observed upon NK cell interaction with the stiffer substrates.

The stiffness properties of a cell undergoes changes during the neoplastic process, with primary tumor cells being stiffer than healthy cells, and conversely metastatic cells showing profound reduction in stiffness (58); in addition, also viral infection increases stiffness by inducing cortical actin rearrangement (59). Thus, we would like to envisage that stiffening or softening would be able to profoundly affect the responsiveness of NK cells infiltrating neoplastic or infected tissues. Because no evidences are presently addressing this issue, we would like to suggest that investigation of mechanotransduction mechanisms in tissue resident NK cells would represent a research area of increasing interest.

## Nanomaterials in the Regulation of NK Cell Functions

The use of nanowires or nanodots functionalized or not with ligands, permits now to explore more in depth the mechanosensitivity of NK cells, and in particular the mechanical features of NKIS. Shaping NK cell activity by nanomaterials to functionally upregulate their activating receptors, may represent an emerging strategy for NK-cell based adaptive immunotherapy. Enhanced NK cell activation on antigen-functionalized and mechanically stimulating nanowires and nanodots, and the isolation of activated NK cell subpopulations, foreruns novel nanoengineered platforms for cell expansion toward therapeutic purposes, with improved efficiency and control of cytotoxic activity (60).

Finally, in the recent years with the development of chimeric antigen receptor (CARs) of T and NK lineages, researchers are closer to achieving high specificity and low off-side effects, with clinical trials achieving remission rates. In this regard, a role of mechanosensing in the antigen/target discrimination is a key to engineered high specific CARs. Further work would be required to completely address the mechanosensing properties of NK cells *in vivo*. These studies can provide important fundamental insights on cytotoxic functions of NK cells and allow rational design of future immunotherapies.

## Concluding Remarks

Cells sense their environment by transducing mechanical stimuli into biochemical signals. Conventional tools for study cell mechanosensing provide limited spatial and force resolution. Recent advances in biomaterials and device engineering have facilitated the generation of numerous artificial cellular microenvironments, which produce synthetic signals mimicking those delivered by the physiological environment. Several advanced materials and devices have been recently produced for the study of mechanical activity in NK cells. NK cells exposed to nanowires functionalized with MICA ligands exhibit higher expression of CD107 degranulation marker, suggesting that the combination of a physical stimulus with the chemical stimulus of MICA, enhances NK cell activation (60, 61). This model may mimic the *in vivo* NK cell/dendritic cell interaction, where the branched projections of DC are similar in size and shape to nanowires (62).

## Author Contributions

GS and AS drafted the manuscript. GS conceived and designed the study. MM, CA, FM, and MS critically revised the manuscript. All authors contributed to the article and approved the submitted version.

## Funding

PRIN-2017 Italian Ministry of Education Ministry of University and Research.

## Conflict of Interest

The authors declare that the research was conducted in the absence of any commercial or financial relationships that could be construed as a potential conflict of interest.
